# Licorice Germplasm Resources Identification Using DNA Barcodes Inner-Variants

**DOI:** 10.3390/plants10102036

**Published:** 2021-09-28

**Authors:** Qianwen Liu, Shuai Guo, Xiasheng Zheng, Xiaofeng Shen, Tianyi Zhang, Baosheng Liao, Wenrui He, Haoyu Hu, Ruiyang Cheng, Jiang Xu

**Affiliations:** 1 Institute of Chinese Materia Medica, China Academy of Chinese Medical Sciences, Beijing 100700, China; L349295748@126.com (Q.L.); tianyizh0530@hotmail.com (T.Z.); swjs082lbs@126.com (B.L.); 2 School of Pharmacy, Chengdu University of Traditional Chinese Medicine, Chengdu 611137, China; shuai93guo@163.com (S.G.); wenrui96017@163.com (W.H.); 3 Institute of Medicinal Plant Physiology and Ecology, School of Pharmaceutical Sciences, Guangzhou University of Chinese Medicine, Guangzhou 510006, China; xszheng@gzucm.edu.cn; 4 Institute of Medicinal Plant Development, Chinese Academy of Medical Sciences, Beijing 100193, China; xfshen1030@163.com

**Keywords:** DNA barcoding, next-generation sequencing (NGS), single-molecule real-time (SMRT) sequencing, germplasm resources, licorice

## Abstract

Based on the gradual transformation from wild growth to artificial cultivation, the accurate authentication of licorice seeds contributes to the first committed step of its quality control and is pivotal to ensure the clinical efficacy of licorice. However, it is still challenging to obtain genetically stable licorice germplasm resources due to the multi-source, multi-heterozygous, polyploid, and hybrid characteristics of licorice seeds. Here, a new method for determining the heterozygosity of licorice seed mixture, based on the various sites, and finding the composition characteristics of licorice seed is preliminarily designed and proposed. Namely, high-throughput full-length multiple DNA barcodes(HFMD), based on ITS multi-copy variation exist, the full-length amplicons of ITS2, *psbA-trnH* and ITS are directly sequenced by rDNA through the next-generation sequence(NGS) and single-molecule real-time (SMRT) technologies. By comparing the three sequencing methods, our results proved that SMRT sequencing successfully identified the complete gradients of complex mixed samples with the best performance. Meanwhile, HFMD is a brilliant and feasible method for evaluating the heterozygosity of licorice seeds. It shows a perfect interpretation of DNA barcoding and can be applied in multi-base multi-heterozygous and polyploid species.

## 1. Introduction

Licorice is one of the most commonly used plant species in Traditional Chinese Medicine (TCM). Its annual demand has exceeded over 37,500 tons since the 1980s [1]. According to the Pharmacopoeia of the People’s Republic of China, the dried radix et rhizoma of the three primary plants, *Glycyrrhiza uralensis* Fisch., *Glycyrrhiza inflata* Bat., and *Glycyrrhiza glabra* L., are now in great demand as medicinal herbs [2]. Some studies have shown significant differences in the content of its active components as well as conspicuous distinctions in efficacy and quality of different basal licorice [3,4]. With the high rate of anthropogenic activities leading to habitat destruction and uncontrolled exploitation, licorice resources have become increasingly severed. With the development of artificial cultivation technology geared towards meeting the growing market demands, cultivated varieties of licorice have become the primary source of licorice medicinal products. Despite this, the quality of the varieties is not up to standard due to various reasons [5,6]. First, there is no gamete isolation in interspecific hybridization of the three licorice varieties, which leads to high hybridization affinity [2,7]. Second, licorice chloroplast DNA is patrilineal, enriching intraspecific genetic diversity and making interspecific hybridization and genetic variation more complex [8]. In addition, the germplasm research of licorice is relatively preliminary. For instance, there are few experienced licorice planting bases, and the selection of various resources is mainly based on breeding, which leads to the complexity of genetic diversity within the population [9,10]. Thus, the accurate identification of the different varieties of licorice is of extraordinary importance to the breeding of TCM and the protection of germplasm resources, which is the source and fundamental problem of TCM production [11]. Hence, it is necessary to effectively identify the seeds of licorice to ensure the efficacy and safety of clinical drug use.

DNA barcoding is a powerful molecular tool for species identification that uses one or several conservative standard DNA sequences in organisms as a marker to identify species [12,13,14]. With the development of the latest cutting-edge technology, TCM research has entered the era of “Herbgenomics”, which reflects the application of DNA barcoding technology in TCM research [15]. Chen S.L. et al. put forward the ITS2 sequence as a generic barcode for medicinal plant identification. They established a botanical medicine DNA barcode identification system with ITS2 as the core and *psbA-trnH* as the supplement sequence for screening multiple DNA barcodes. This approach has laid a solid foundation for developing DNA barcoding technology to identify Chinese medicine [16]. In the practical application of licorice, DNA barcoding is mainly used for phylogenetic classification and species identification of plant species [17,18]. In addition, DNA barcoding based on ITS2 and *psbA-trnH* has been successfully used for the molecular identification of medicinal plants seeds [19,20,21,22]. Nevertheless, its application in germplasm resources of multi-species origin Chinese medicine is rarely involved. It is challenging to identify the germplasm resources of licorice due to its high genetic diversity, complex intraspecific populations, and the presence of multiple seeds mixing [23,24].

Given the advancement in science and technology, Next-generation Sequencing (NGS) and single-molecule real-time (SMRT) technology have emerged as potential approaches in taxonomy. This is due to the advantages of longer sequencing reads and deeper sequencing depth [25,26]. Long-read sequencing techniques have already been used in some aspects. They have furnished novel guidelines and ways for identifying Chinese patent medicines and formulae, which is difficult due to many complex mixtures [27]. Full-length multi-barcoding (FLMB) technique of long-read sequencing was proposed to identify biological components in TCM compounds by direct sequencing of ITS2 and *psbA-trnH* full-length amplicon using SMRT technology [28]. In addition, ribosomes and chloroplasts are abundant organelles in living cells, which means that there are numerous copies of ITS2 and psbA-trnH sequences [29,30]. Several studies have shown that the repeats are not identical, with variability within the intergenic regions that may be the breakthrough point for the identification and screening of licorice seeds [31,32,33]. All these provide references for the screening of licorice germplasm resources.

**Scheme 1:**
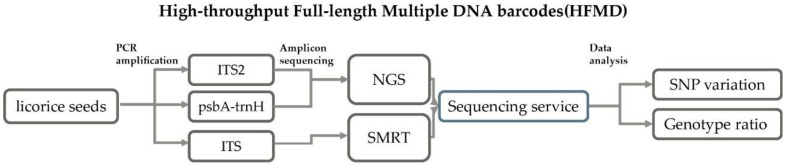
The pipeline of HFMD. HFMD for licorice seeds mainly includes PCR amplification, amplicon sequencing, and data analysis.

Herein, a novel strategy (as shown in Scheme 1) was proposed to sequence and analyze the seed samples of licorice using NGS and SMRT approaches to supplement the DNA barcode adhibition of the germplasm resources of licorice. The strategy can evaluate the internal heterozygosity of licorice group seed samples and single seed samples to screen reliable germplasm resources and explore the variation in the characteristic sites of licorice, which can be used as the basis for subsequent experiments to examine its rules further. To a certain extent, this method is also of great significance for heterogeneity identification of multiple samples and screening Chinese medicine germplasm resources with analogous heterozygous copy variation or interspecific hybridization. The extension of DNA barcoding technology application to germplasm resources filtration of Chinese medicinal materials can not only guarantee the accuracy of planting species from the source, but also be of great significance for promoting the standardization of germplasm resources [11].

## 2. Results

### 2.1. Morphological Characteristics of Licorice Seeds

**Figure 1:**
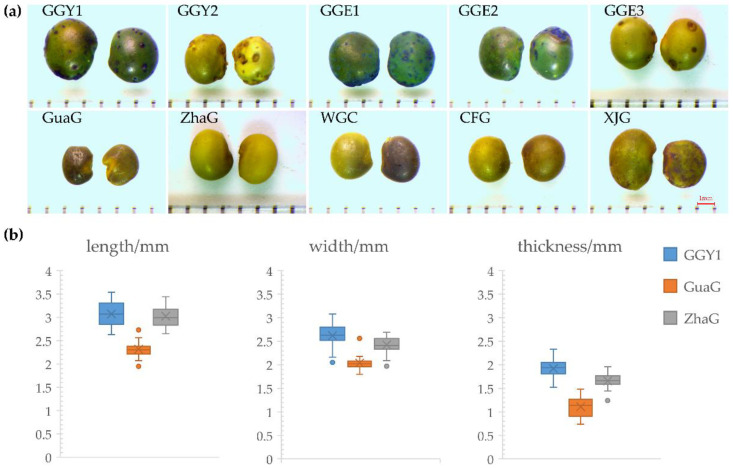
Morphology and size statistics box diagram of licorice seeds. (**a**) Morphology of ten samples of licorice seeds. One grid below the seed’s ruler indicates 1 mm. (**b**) Statistical graphs of the length, width, and thickness of 30 licorice seeds, respectively. Different colored boxes represent other species: the blue box is sample GGY1, representing *G. uralensis*; the orange box is sample GuaG, representing *G. glabra*; and the grey box is sample ZhaG, representing *G. inflata*.

The sample morphology and measurement results of the length, width, and thickness of the three kinds of licorice seeds are shown in Figure 1. The seeds of *G. uralensis* are round, kidney-shaped, and slightly flattened, with significantly variations in size (2.63~3.54 mm in length, 2.05~3.08 mm in width, and 1.52~2.33 mm in thickness). These seeds also vary greatly in the episperm color, brown-green, light yellow, light black, dark green, dark yellow, deep black, or mostly with brown-green, and even the same seed coat color shades are diverse.

The seeds of *G. glabra* were the smallest in size, oblate and round, and varied slightly in size as follows: 1.95~2.73 mm long, 1.80~2.56 mm wide, and 0.74~1.48 mm thick. The episperm color is brownish yellow to brownish brown.

The seeds of *G. inflata* are full and round kidney-shaped, with a length of 2.65~3.44 mm, a width of 1.97~2.69 mm, and a thickness of 1.24~1.96 mm. The surface is greyish yellow or light brownish-green, smooth, and slightly shiny. Other characteristics are similar to those of *G. uralensis*.

Compared to *G. uralensis*, the seed size and color of the seed coat are more varied. For instance, *G. glabra* and *G. inflata* are more uniform in seed size and episperm color. The seeds of *G. glabra* are flat and round in shape and are the smallest in size compared to the other two. The seeds of *G. uralensis* and *G. inflata* are similar in size, although with some differences. For example, the latter is lighter in color and plumper in shape. However, it was difficult to distinguish the seeds of *G. inflata* based on morphology when mixed with the seeds of the other licorice varieties.

### 2.2. Sanger Sequencing of DNA Barcoding

The sequence analysis result showed that the length of the ITS2 sequence of licorice is 223 bp. There are three mutation sites at 16~18 bp, which can be divided into two genotypes: I2-i (TGC) and I2-ii (CAA), of which the former is the haplotype of *G. uralensis* and the latter is the haploty pe of *G. glabra* and *G. inflata*. The length of the ITS sequence is 618 bp, with four mutation sites at 187 bp and 411~413 bp (the same position as 16~18 bp in ITS2 sequence), respectively, which could be divided into two genotypes: I-i 187C-411T-412G-413C, and I2-ii (187T-411C-412A-413A), of which the former is the haplotype of *G. uralensis* and the latter is the haplotype of *G. glabra* and *G. inflata*. No sequence variations were observed for all the ITS2 and ITS amplicons of samples with the same species. In ITS2 sequence also ITS sequence, the sequencing results of the remaining eight samples were i (I2-i for ITS2, I-i for ITS), except for two samples whose haplotype were ii (I2-ii for ITS2, I-ii for ITS). The length of the *psbA-trnH* sequence of licorice is 305 bp, with three variation loci located at 189 bp, 235 bp, and 288 bp, respectively. The haplotypes can be classified into four: PT-i (189A-235C-288G), PT-ii (189A-235T-288G), PT-iii (189C-235T-288G), and PT-iv (189A-235T-288A). Among them, haplotypes PT-i and PT-ii are unique to *G. uralensis*, haplotype PT-iii appears in *G. glabra*, and haplotype PT-iv is unique to *G. inflata*. Four samples were identified as PT-i and PT-ii, and one sample was identified as PT-iii and PT-iv. The haplotype distribution of sequences is shown in Table 1.

**Figure 2:**
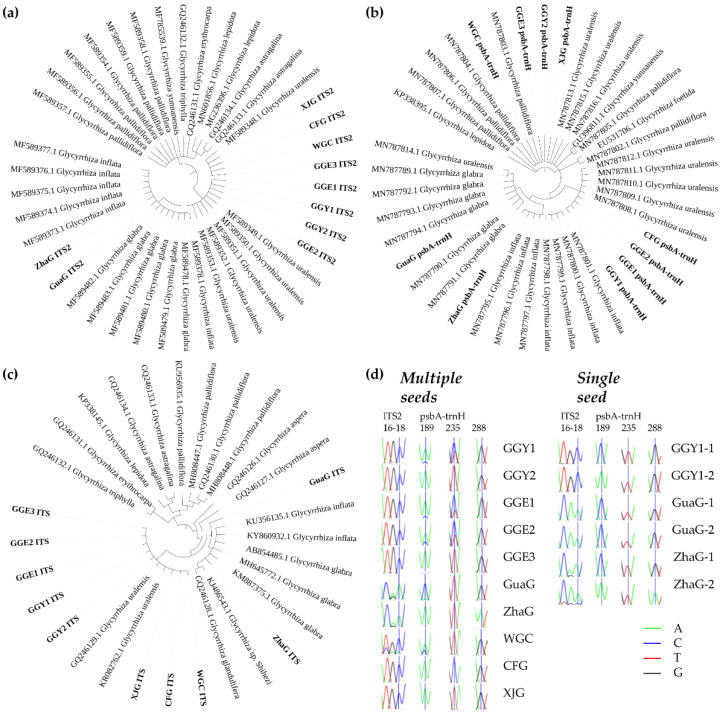
Phenetic tree and chromas file of Sanger sequencing identification results. (**a**–**c**) are the phylogenetic tree of ITS2, *psbA-trnH*, and ITS sequences of Glycyrrhiza species, respectively. Sample sequences sequenced by Sanger are shown in bold, and the remaining sequences came from GenBank, preceded by the accession number. (**d**) From left to right are the sanger sequencing peaks of mutation sites in multiple seeds and a single seed, respectively. Each nucleotide is color-coded: A for adenine in green, T for thymine in red, C for cytosine in blue, and finally G for guanine in black.

Meanwhile, we found that there were nesting peaks in the chromas file of the sequencing results. Additionally, there were different base nesting peaks in the mutation sites of some samples. The verification experiment carried out with individual seeds of GGY1, GuaG, and ZhaG showed the existence of nesting peaks (Figure 2d). This indicates that nested peaks are not only due to mixed seeds but, more importantly, the multiple copy variation in rDNA samples and the deviations of individual variation. In the chromas file, it can be found that there is no nesting peak in the two seed samples of *G. glabra*. An interlocking peak appeared in both the two seeds of *G. inflata* (site 17 and site 16~18, respectively), while a nesting peak appeared in one of the seed samples of *G. uralensis* (site 16), and no nesting peak appeared in the other. Furthermore, the gene tree analysis based on the ITS2 sequence and ITS sequence showed that *G. uralensis* formed a distinct clade, as it does not cluster in the same branch with *G. glabra* and *G. inflata*. However, *G. glabra* and *G. inflata* clustered in the same branch and cannot be distinguished. Using the *psbA-trnH* sequence, the three species are on different branches. The *G. uralensis* is divided into two sub-clades that correspond to the two different haplotypes of *G. uralensis* (Figure 2a–c). The results showed concatenated ITS2+*psbA-trnH* can easily delineate the three varieties of licorice.

### 2.3. Full-Length Amplicon Sequencing of ITS2, psbA-trnH, and ITS

**Figure 3:**
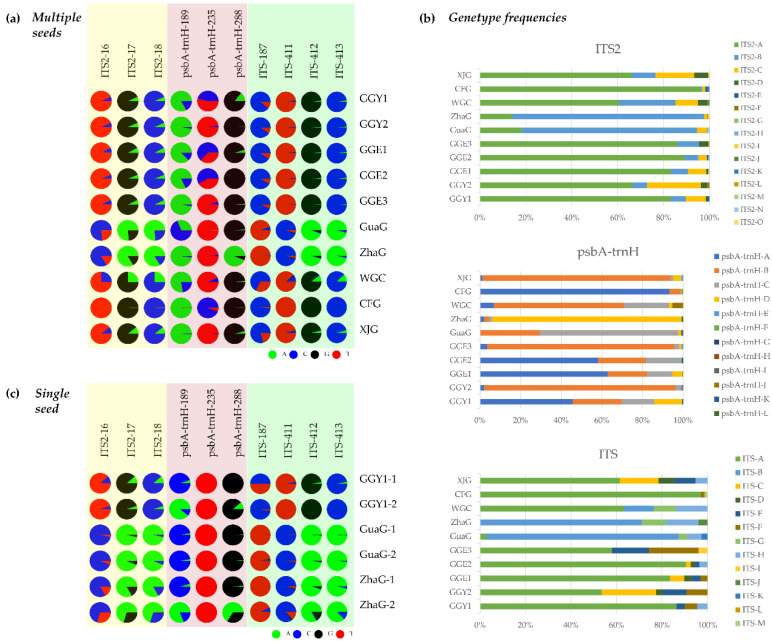
Percentage frequency of each base of genotype and mutation site. (**a**,**c**) The statistics of frequency percentage of each base of the variation site in multiple seeds and single seed samples, respectively. Yellow, pink, and cyan indicate molecular markers ITS2, *psbA-trnH*, and ITS. Each nucleotide is color-coded: A for adenine in green, T for thymine in red, C for cytosine in blue, and finally G for guanine in black. The matrix pie chart was plotted by http://www.bioinformatics.com.cn, accessed on 13 August 2021, an online platform for data analysis and visualization. (**b**) The frequencies of different genotypes in molecular markers ITS2, *psbA-trnH*, and ITS of multiple seeds, from top to bottom.

To verify whether the repeats with a variation that appeared in Sanger sequencing are accurate, amplicons of ITS2, *psbA-trnH*, and ITS were sequenced (Table 2). After alignment process, the sequences statistical analysis results showed that, in addition to the genotypes mentioned in Table 1, there are other different genotypes, as shown in Figure 3b. The differences among the ten samples are significant, and the dominant genotypes of *G. glabra* and *G. inflata* are different from *G. uralensis*. The results also reflect the existing internal heterozygosity among licorice seeds.

The statistical analysis results of the distribution bases showed SNPs existed in the licorice samples, which were placed in the dominant or secondary bases according to the ratio of bases, as shown in Figure 3. Results of the Sanger sequencing and HFMD sequencing were consistent. In the latter results, when SNP secondary bases accounted for a large proportion, nested peaks were shown in the chromas file; when SNP secondary bases accounted for a small proportion, unimodal peaks were observed. For ITS2 and ITS, the proportion of secondary bases at the three consecutive mutation sites was consistent. In comparison, the proportion of secondary bases at the three discontinuous mutation sites of *psbA-trnH* were considerably different. This suggested that variations in the three contiguous sites are correlated, but not in the discontinuous sites. However, we do not know what causes the correlation. The percentage and frequency of non-dominant genotype in the CFG sample were the lowest in any molecular marker, indicating that the heterozygosity of its seed population was the lowest in the licorice seed sample.

To investigate whether the interpeak was introduced due to the impure mix of samples, we performed the same sequencing on two single seeds from each of the three samples. All single seeds were morphologically identified to ensure their species. The results showed that the primary and secondary bases on the interpeak and mutation sites were also present in the first-generation sequencing results of a single seed sample. The results showed that the proportion of the two seeds of the same species was not consistent. Moreover, compared with the base proportion of the multiple mixed samples, the variation proportion of the single seed sample and the seed population sample was also different. These results indicate that the variation in the seed population is likely to be caused by either the variation within a single seed or the mixing of multiple seeds with different variations. There are still differences in the proportion of each genotype in single seed samples and mixed seeds.

## 3. Discussion

DNA barcoding was first proposed by Canadian zoologist Hebert and gradually improved with the active exploration and supplementation of scholars at home and abroad [34,35,36,37,38]. The Consortium for the Barcode of Life (CBOL) has recommended the chlorophyte gene rbcL, matK, *psbA-trnH*, and the nuclear gene ITS as standard DNA barcodes for land plants, combined with the results of analysis of data from previous studies [39]. As a cost-effective, standardized approach for rapid species identification, DNA barcoding has been widely used in almost all types of organisms, extensively promoting the development of modern Chinese medicine identification [40,41]. The successful establishment and widespread application of the TCM species barcode identification in Global Pharmacopoeia Genome Database (http://www.gpgenome.com:8080/, accessed on 13 August 2021) [42,43], which can quickly and easily perform species identification, successfully supplemented the traditional identification methods such as trait identification [44], microscopic identification, and physicochemical identification, and has brought new opportunities for the species identification of seeds and seedlings of Chinese medicinal materials [45,46].

As one of the most commonly used traditional Chinese medicine, the demand for licorice is increasing day by day, and artificial cultivation is increasingly needed to meet the needs of clinical medicine [1,5]. However, due to the polyploidy of the base source and the incomplete isolation between species, there are many heterozygous features, and it is difficult to screen stable genetic germplasm for large-scale cultivation, which also causes frequent segregation of progeny traits in the existing licorice planting process. There is no unified quality medicinal materials market, leading to an erratic clinical treatment of licorice [8,24,47]. Morphological and molecular identification of ten licorice seed samples was carried out. In terms of morphological identification, only G. glabra seeds can be easily distinguished. In contrast, *G. uralensis* and *G. inflata* seeds are indistinguishable due to the high overlap of color, shape, and size (Figure 1). The Sanger-sequenced DNA barcode (ITS2+*psbA-trnH*) can be used to identify the three licorice in terms of molecular identification (Figure 2a–c). However, after careful analysis of the identification results, it was found that there were nested peaks in the results of the first-generation sequencing chromas file, and the degree of hybridization within the seeds could not be determined (Figure 2d).

To verify whether the sleeve peak exists, and to explore the reasons for its appearance, we used NGS and SMRT techniques to perform high-throughput sequencing on high-throughput full-length multiple DNA barcodes based on rDNA with multiple copies of variation [28,32]. Sequencing results confirmed the actual existence of nesting peak; that is, there are different genotypes in the samples, and additional samples have different genotypes and proportions (Figure 3b). The results showed that the proportion of the two seeds of the same species was not consistent. Moreover, compared with the base proportion of the multiple mixed seeds of the sample, the variation proportion of the single seed sample and the seed population sample was also different. In individual sample GGY1-1, the morphology was identified as *G. uralensis*. In molecular identification, haplotype I2-i in ITS2 was identified as *G. uralensis*. At the same time, PT-iii in *psbA-trnH* of chloroplast pointed to *G. glabra*, and nearly half of the 187 sites in ITS were secondary bases (Figure 2d and Figure 3c). In the individual sample ZhaG-1, the morphological identification was *G. inflata*, yet haplotype PT-iii in *psbA-trnH* of chloroplast pointed to *G. glabra*. Surprisingly, no haplotype PT-iii in *psbA-trnH* of *G. inflata* has been reported [48,49,50,51]. Therefore, it is speculated that the hybridization event occurred in the generation of sample GGY1-1 and ZhaG-1 with a male parent of *G. glabra*, which is based on the characteristic that the chloroplast DNA of glycyrrhiza interspecific hybridization is mainly inherited by the male parent [8,52]. To obtain stable genetic single glycyrrhiza germplasm resources, the low heterozygosity of the seed population should be ensured, and the low variability of the single seed in the population should be confirmed. The wild seeds from Chifeng, Inner Mongolia, China, showed low heterozygosity and enormous breeding potential, whether in proportion statistics of genotypes or base SNP sites in ten seed population samples (Figure 3a,b). Nonetheless, further identification and research of more individual samples are still needed to proceed by HFMD to determine the variation of molecular markers in seeds accurately.

In addition, compared to NGS and SMRT sequencing results, we think that Sanger sequencing will hide the SNP phenomenon. If the proportion of SNP base variation is relatively low, the latter cannot distinguish the variation of the lower peak graph and consider it as noise interference. Only when the proportion of SNP base variation is relatively high can it be ignored and displayed in the form of a peak. Suppose the SNP variation of the base is lower. In that case, generation sequencing results cannot distinguish a low peak in variation, and note it as noise interference. Only the SNP variation of the base is higher, cannot be ignored, and will be displayed in the form of nested peaks [53,54,55]. By comparing Sanger sequencing and SMRT sequencing results, we believe that NGS will amplify the base proportion of SNP sites and cause false positives, which may be due to the need for repeat PCR amplification within it, which is related to the bias of PCR amplification [32,56,57].

In this study, a novel method, HFMD, has been successfully developed to evaluate the internal heterozygosity of licorice group seed samples and single seed samples by NGS and SMRT sequencing to screen reliable germplasm resources and explore the variation in the characteristic sites of licorice, which can be used as the basis for subsequent experiments to examine its rules further. An objective comparison of the three sequencing methods was made, and the results showed that SMRT sequencing results were relatively accurate, as Sanger sequencing would cover up the SNP phenomenon, while the preference of PCR in the NGS would amplify the proportion of the secondary bases of the SNP site. It has important implications for the selection of other sequencing methods. To a certain extent, this method also provides a direction for heterogeneity identification of multiple samples and screening Chinese medicine germplasm resources with similar heterozygous copy variation or interspecific hybridization.

## 4. Materials and Methods

### 4.1. Sample Collection of Licorice Seeds

Ten licorice seed samples were collected from medicinal materials markets and production places, which contained two batches of market A; three batches of market B; one batch each from market C for pure *G. glabra*, pure *G. inflata*, and *G. uralensis*; and two batches of wild samples from Chifeng City, Inner Mongolia Autonomous Region, China and Xinjiang Autonomous Region, China, respectively (Table 3). All materials were identified by their morphologies.

### 4.2. Total DNA Extraction, PCR Amplification and Sanger Sequencing

We took samples of multiple fresh seeds and single seeds and ground them into powder. Total DNA was extracted using the Plant Genomic DNA Kit (Tiangen Biotech Co., Ltd., Beijing, China). PCR systems contain 1 × Taq MasterMix (Aidlab Biotechnologies Co., Ltd., Beijing, China), 1 μM of each primer, and ~100 ng DNA templates. The PCR primers and conditions used to amplify each barcode region are shown in Table 4. Sanger sequencing of those PCR products were provided by Tsingke Biotechnology Co., Ltd. Beijing, China. Codoncode Aligner V5. 1.5 (CodonCode Co., Centerville, MA, USA) software was used to calibrate and splice the sequencing results, and the low-quality sequences and primer regions were removed. Clustal W alignment and Neighbour-joining Tree (NJ Tree) were performed using MEGA 6.0 to confirm their biological origin.

### 4.3. NGS and SMRT Sequencing

Amplification, sequencing, and analysis were performed according to the procedure shown in Scheme 1. All DNA samples were used as templates for PCR amplification of ITS2, *psbA-trnH*, and ITS, respectively. Amplification for each amplicon was carried out using different pairs of tag primers, to which several protective bases and labelling bases were attached to the 5′ end of the conventional primers. The ITS2 and the *psbA-trnH* amplicon was sequenced by Illumina Miseq PE300 at a depth of no less than 30 thousand pieces per sample, while ITS amplicon using PacBio Sequel. Sequencing service was provided by Personal Biotechnology Co., Ltd. Shanghai, China.

### 4.4. Sequencing Results and Data Analysis

All sequencing results are quality controlled. The sample sequences of ITS2, psbA-trnH and ITS amplicons of 10 licorice samples were aligned by the Burrows–Wheeler Aligner-Minimum Exact Match (BWA-MEM) (v 0.7.17). After alignment process, the sequences statistical analysis results the statistical analysis was conducted on the sequencing results. A statistical sample of the same molecular marker in the presence of different genotypes and their proportion was carried out. In addition, the proportion of each base on the SNP site in the molecular marker of the sample was also statistically analyzed to determine heterozygosity through molecular identification.

## 5. Conclusions

In this paper, a novel idea for estimating the heterozygosity of licorice seeds via NGS and SMRT sequencing technology was preliminarily established, and the copy variation characteristics inside the SNPs of licorice were explored, which was of great significance for the screening of the Chinese medicine germplasm resources with analogous heterozygous copy variation. In addition, the three sequencing methods are compared objectively, which can be used for reference in the selection.

## Figures and Tables

**Table 1:** Variation loci of licorice in three molecular markers and identification results of seed samples.

**1. ITS2**	variable site/bp				
haplotype	16	17	18	Species	Sample
I2-i	T	G	C	*G. uralensis*	GGY1, GGY2, GGE1, GGE2, GGE3, WGC, CFG, XJG
I2-ii	C	A	A	*G. glabra*/*inflata*	GuaG, ZhaG
**2. ITS**	variable site/bp				
haplotype	187	411~413		Species	Sample
I-i	C	TGC		*G. uralensis*	GGY1, GGY2, GGE1, GGE2, GGE3, WGC, CFG, XJG
I-ii	T	CAA		*G. glabra*/*inflata*	GuaG, ZhaG
**3. *psbA-trnH***	variable site/bp				
haplotype	189	235	288	Species	Sample
PT-i	A	C	G	*G. uralensis*	GGY1, GGE1, GGE2, CFG
PT-ii	A	T	G	*G. uralensis*	GGY2, GGE3,WGC, XJG
PT-iii	C	T	G	*G. glabra*	GuaG
PT-iv	A	T	A	*G. inflata*	ZhaG

**Table 2:** Processing information for HFMD sequencing.

Total Reads	Raw Data	Merged	Filtered	Mean Length/bp
**ITS2**	1,387,176	1,246,275	1,199,501	407
***psbA-trnH***	1,128,832	1,120,013	1,082,034	389
	**Sub Reads**	**Circular Consensus Sequence Filtration**		**Mean Length/bp**
**ITS**	14,540,927	218,721		736

**Table 3:** Information on ten samples of licorice seeds.

Sample	Organ	Specie	Wild/Cultivated	Description
GGY1	Seeds	*G. uralensis*	Cultivated	batch 1 of Market A
GGY2	Seeds	*G. uralensis*	Cultivated	batch 2 of Market A
GGE1	Seeds	*G. uralensis*	Cultivated	batch 1 of Market B
GGE2	Seeds	*G. uralensis*	Cultivated	batch 2 of Market B
GGE3	Seeds	*G. uralensis*	Cultivated	batch 2 of Market B
GuaG	Seeds	*G. glabra*	Wild	Market C
ZhaG	Seeds	*G. inflata*	Wild	Market C
WGC	Seeds	*G. uralensis*	Wild	Market C
CFG	Seeds	*G. uralensis*	Wild	Chifeng, Inner Mongolia, China
XJG	Seeds	*G. uralensis*	Wild	Xinjiang, China

**Table 4:** The PCR primers and conditions used to amplify each barcode region.

Region	Primer Sequence	Program
**ITS2**	ITS2-P3: YGACTCTCGGCAACGGATA ITS2-E4: RGTTTCTTTTCCTCCGCTTA [44]	94 °C for 5 min; 94 °C for 1 min, 55 °C for 1 min, 72 °C for 1.5 min, 30 cycles; and 72 °C for 7 min
***psbA-trnH***	psbAF: GTTATGCATGAACGTAATGCTC trnHR: CGCGCATGGTGGATTCACAATCC [16]	94 °C for 5 min; 94 °C for 1 min, 55 °C for 1 min, 72 °C for 1.5 min, 30 cycles; and 72 °C for 7 min
**ITS**	ITS4:TCCTCCGCTTATTGATATGC ITS5:GGAAGTAAAAGTCGTAACAAGG [58]	95 °C for 3 min; 95 °C for 30 s, 55 °C for 30 s, 72 °C for 1.5 min, 35 cycles; and 72 °C for 10 min

## Data Availability

Publicly available datasets were analyzed in this study. This data can be found here: [*G. glabra*: http://www.gpgenome.com/species/436, accessed on 13 August 2021; *G. inflata*: http://www.gpgenome.com/species/437, accessed on 13 August 2021; *G. uralensis*: http://www.gpgenome.com/species/438, accessed on 13 August 2021].
